# Unveiling of Type B Lactic Acidosis From Systemic Lupus Erythematosus-Associated B-cell Lymphoma: A Fatal Oncology Emergency

**DOI:** 10.7759/cureus.38648

**Published:** 2023-05-06

**Authors:** Yashitha Chirumamilla, Nageshwari Palanisamy, Ekwevugbe Ochuko O Ogbon, Justine Chinnappan, Terry Krznarich, Ghassan Bachuwa, Mohammed Berrou

**Affiliations:** 1 Internal Medicine, Hurley Medical Center, Flint, USA; 2 Pathology, Hurley Medical Center, Flint, USA; 3 Pulmonary and Critical Care Medicine, Hurley Medical Center, Flint, USA

**Keywords:** oncological emergency, type b lactic acidosis, refractory lactic acidosis, diffuse lymphadenopathy, diffuse large b lymphoma, acidosis lactic

## Abstract

Type B lactic acidosis is a rare oncological emergency usually associated with leukemia and lymphoma but also with solid malignancies. It can often go unrecognized as a possible source of lactic acidosis, leading to a delay in treatment. We review a 56-year-old woman with systemic lupus erythematosus and generalized lymphadenopathy being evaluated for underlying malignancy who presented with dyspnea, fatigue, and hematemesis. The patient was hemodynamically unstable and had severe lactic acidosis, leukocytosis, electrolyte derangements, multiple organ damage, and worsening diffuse lymphadenopathy. She was initially treated for septic shock due to acalculous cholecystitis on imaging with antibiotics and a cholecystostomy. The latter was complicated by a liver laceration requiring explorative laparotomy and open cholecystectomy, during which an excisional biopsy of the omental lymph node was done and confirmed B-cell lymphoma with marked plasmacytic differentiation. Her lactic acidosis never fully cleared despite surgery, and the refractory nature of it despite appropriate treatment of septic shock confirmed the diagnosis of type B lactic acidosis from underlying B-cell lymphoma. Chemotherapy was deferred due to the acuity of the condition. She continued to deteriorate despite aggressive management and was transitioned to comfort measures only per family request, following which she passed away.

Type B lactic acidosis should be suspected in oncology patients without clinical evidence of ischemia who are not responding to fluid resuscitation and appropriate treatment of septic shock. Prompt recognition and early initiation of antineoplastic agents should be considered, when possible, to prevent adverse outcomes.

## Introduction

Lactic acidosis occurs with severe hyperlactatemia (>5mmol/L) and is associated with increased anion gap metabolic acidosis. It is broadly classified into types A and B [1]. Type A is the most common and is associated with decreased tissue perfusion and hypoxia caused by various types of shock. Type B is comparatively rare yet lethal and occurs in the absence of hypoperfusion and hypoxia. It is further subdivided into type B1, which is disease-associated, including malignancy, thiamine deficiency, diabetic ketoacidosis, liver failure, and renal failure. Type B2 is drug- or toxin-associated, and type B3 is caused by inborn errors of metabolism [2]. Lactic acidosis can sometimes present as a mixture of types A and B as well, which becomes a challenge for diagnosis. In any case, refractory lactic acidosis requires further investigation to identify the underlying cause and initiate prompt treatment.

This article was previously presented as a poster presentation at the American College of Physicians Michigan Chapter 2022 Annual Scientific Meeting held in Bellaire, Michigan, on October 14, 2022.

## Case presentation

A 56-year-old woman with systemic lupus erythematosus complicated by lupus pneumonitis and jugular vein thrombosis, hypertension, anemia of chronic disease, and immune thrombocytopenic purpura requiring splenectomy presented to the emergency department with complaints of abdominal pain, hematemesis, dyspnea, and fatigue. She was recently evaluated for generalized lymphadenopathy associated with weight loss, with a core needle biopsy revealing atypical lymphoid infiltration of the right axillary lymph node. Hence, lymph node excision was planned for a definitive diagnosis, but she presented prior to that with the above-mentioned symptoms. On presentation, she was hemodynamically unstable with a blood pressure of 79/44 mmHg, a heart rate of 150 beats per minute, and a respiratory rate of 60 breaths per minute. Physical examination was significant for ill appearance, confusion, cachexia, dry mucous membranes, and dry blood in the mouth. Laboratory workup revealed lactic acidosis with a pH of 7.09, low bicarbonate, and elevated lactate levels. The patient had a low hemoglobin, an elevated white blood cell count, and a low platelet count. Further laboratory values included elevated lactate dehydrogenase (LDH), transaminitis, acute kidney injury, and elevated prothrombin time and international normalized ratio (INR) (Table 1).

**Table 1:** Initial laboratory values upon presentation to the emergency department. AST: aspartate transaminase; ALT: alanine transaminase; BUN: blood urea nitrogen; PT: prothrombin time; INR: international normalized ratio

Laboratory marker	Values on presentation	Reference range
Venous pH	7.09	7.30 -7.40
Venous bicarbonate	5.4	22 - 28 mEq/L
Lactate	17.8	0.5 - 2.2 mmol/L
White blood cell count	14.0	4 - 10.8 k/uL
Hemoglobin	6.8	12 -16 g/dL
Platelet count	70	130 - 430 k/uL
Lactate dehydrogenase	970	100 - 225 U/L
Alkaline phosphatase	247	30 - 143 U/L
AST	54	0 - 40 U/L
ALT	31	7- 40 U/L
BUN	73	6 - 20 mg/dL
Creatinine	3.3	0.5 - 1.1 mg/dL
PT	22.4	12 - 14.7 seconds
INR	2.06	< 4.0

**Figure 1:**
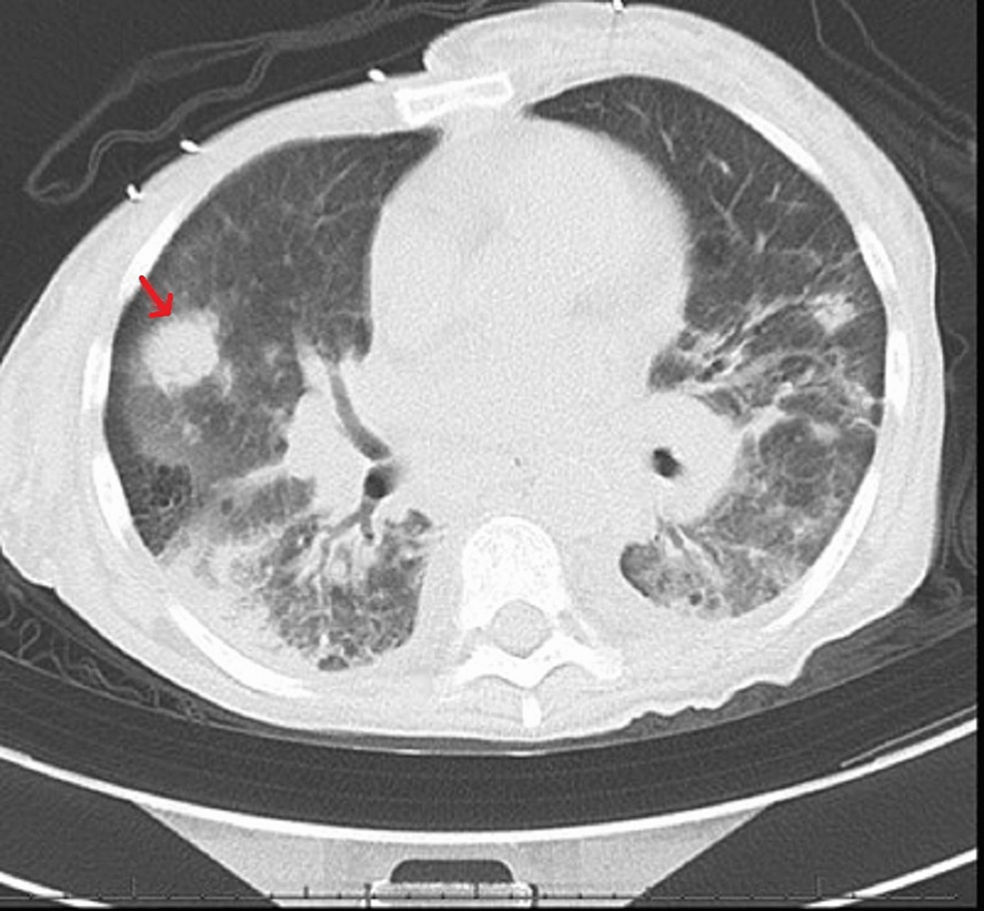
Computed tomography of the chest (axial view) showing a pulmonary nodule in the right middle lobe (red arrow) and bibasilar fibrotic changes.

**Figure 2:**
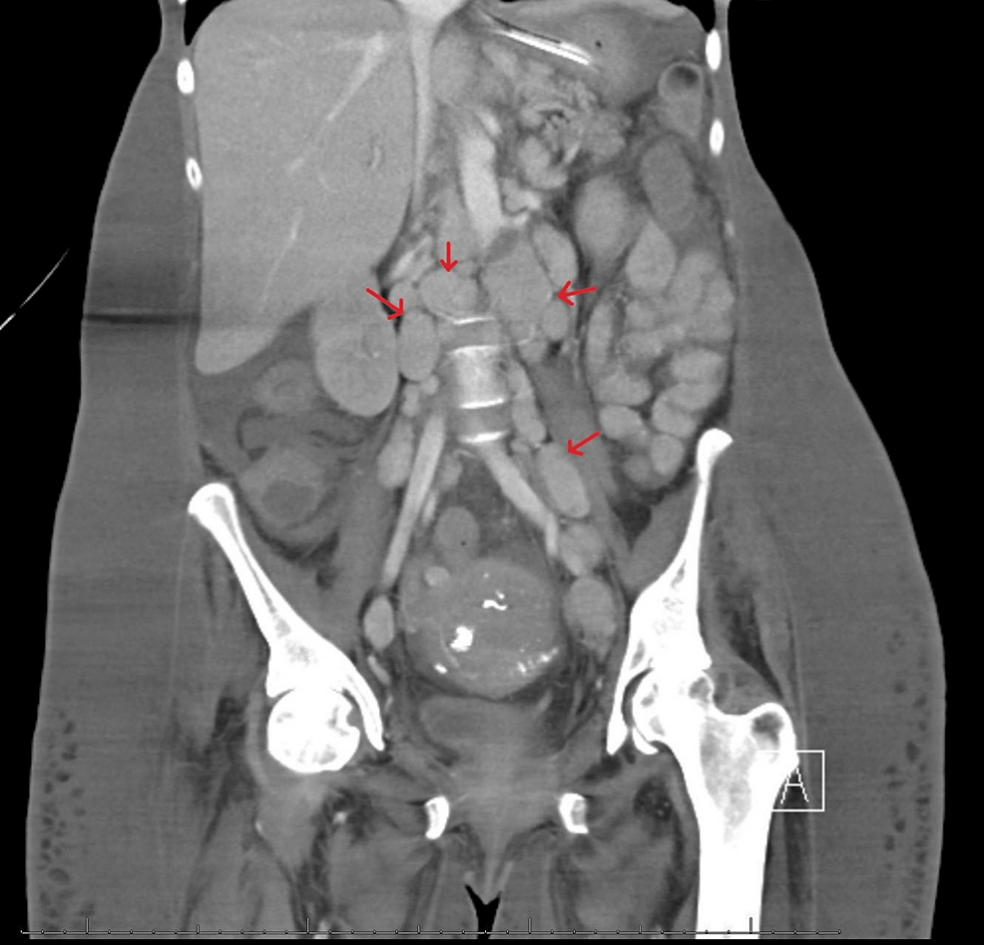
Computed tomography of the abdomen (coronal view) showing multiple enlarged retroperitoneal lymph nodes (red arrows).

**Figure 3:**
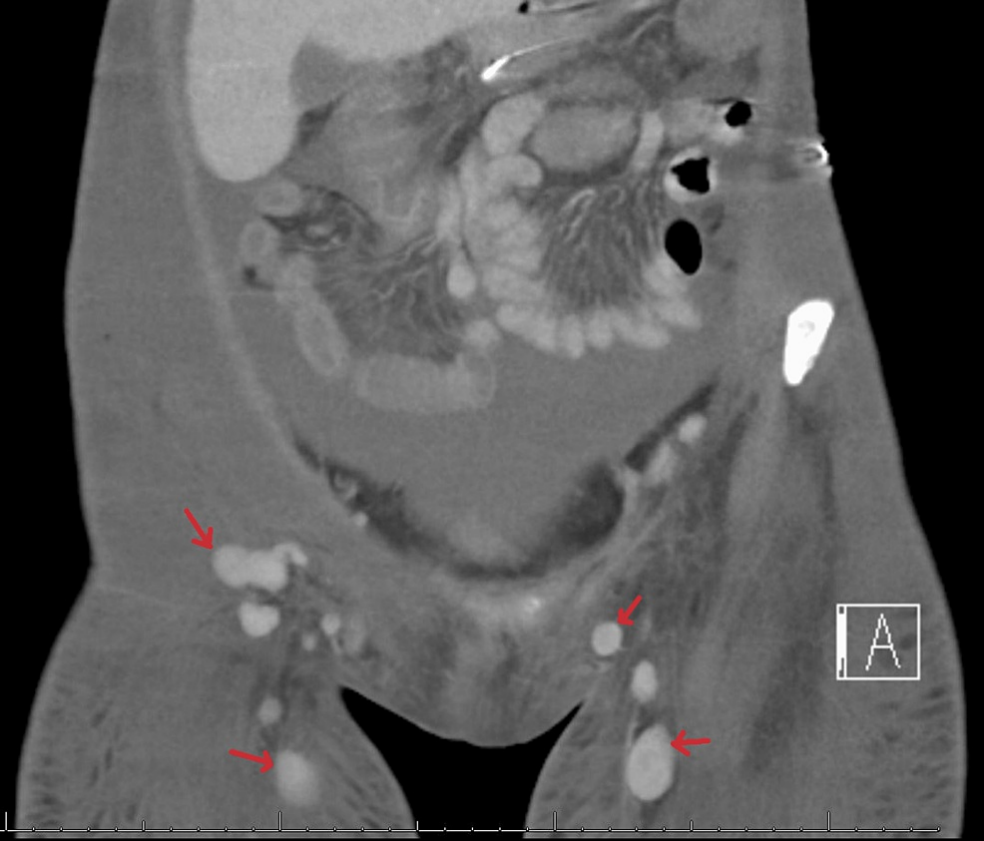
Computed tomography of the abdomen (coronal view) demonstrating bilateral inguinal lymphadenopathy (red arrows).

**Figure 4:**
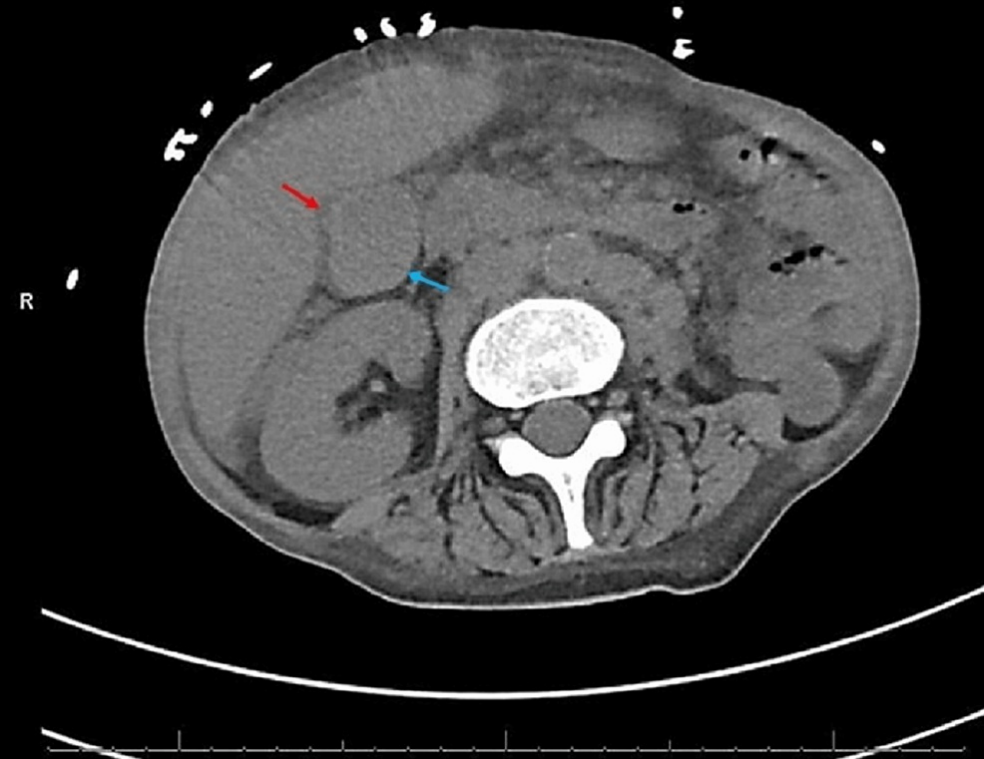
Computed tomography of the abdomen (axial view) showing a distended gallbladder (blue arrow) with a thickened wall and pericholecystic fluid (red arrow).

Computed tomography (CT) showed multiple new pulmonary nodules compared to prior imaging, worsening diffuse lymphadenopathy, and a distended thick-walled gallbladder with pericholecystic fluid concerning acute acalculous cholecystitis (Figures 1-4).

The initial working differential diagnosis was septic shock secondary to acalculous cholecystitis, substantiated by leukocytosis, hemodynamic instability, and cholecystitis on imaging. Hemorrhagic shock was also considered due to hematemesis, low hemoglobin associated with hemodynamic instability, and evidence of coagulopathy.

**Figure 5:**
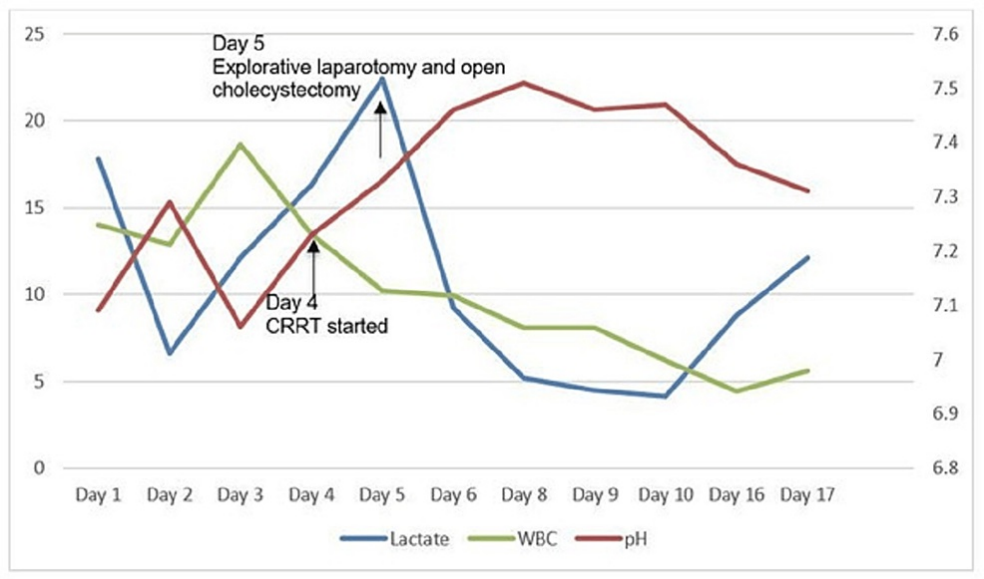
Trend of lactic acid, pH, and white blood cell count during her hospital course. Lactic acid is found to downtrend following surgery before trending up again and never reaching a value of <2 mmol/L. Leukocytosis, on the other hand, resolved following definitive treatment for sepsis. *The pH of days one and two are from venous blood, while the rest is from arterial blood. The X-axis represents the day of the hospital stay. The primary Y-axis on the left represents the white blood cell count in K/uL. The secondary Y-axis on the right represents pH. WBC: white blood cell count; CRRT: continuous renal replacement therapy

She was managed with broad-spectrum antibiotics, intravenous esomeprazole, blood product transfusions, and intravenous fluids. However, she deteriorated hemodynamically with hypotension, metabolically with worsening renal function and acidosis, and clinically with declining mentation, requiring mechanical ventilation, pressor support, sodium bicarbonate infusion, and continuous renal replacement therapy (CRRT). She underwent an ultrasound-guided cholecystostomy with drain placement, which was complicated by hemoperitoneum. She eventually required an emergent exploratory laparotomy, which revealed bleeding from the liver with the cholecystostomy tube entering the liver parenchyma. Hemostasis was achieved intraoperatively; cholecystectomy and excisional biopsy of multiple omental lymph nodes were performed. Following the surgery, she had improvement in lactic acidosis and was weaned down to one pressor support, but her lactate level never normalized (Figure 5). She continued on broad-spectrum antibiotics as well as antifungals, though her blood and bile cultures remained negative.

**Figure 6:**
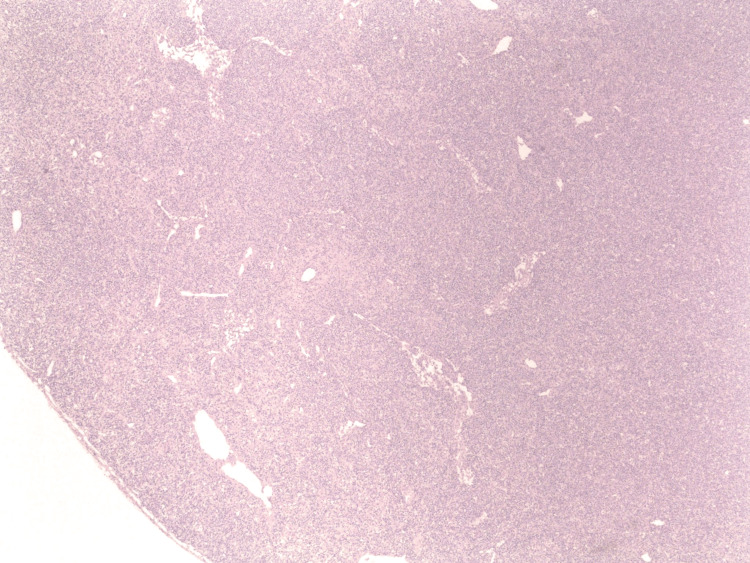
Histopathology findings on H&E stain of the omental lymph node showing complete distortion of the lymph node architecture due to diffuse infiltration of the atypical lymphocytes. Cortical sinuses are patent (20x magnification).

**Figure 7:**
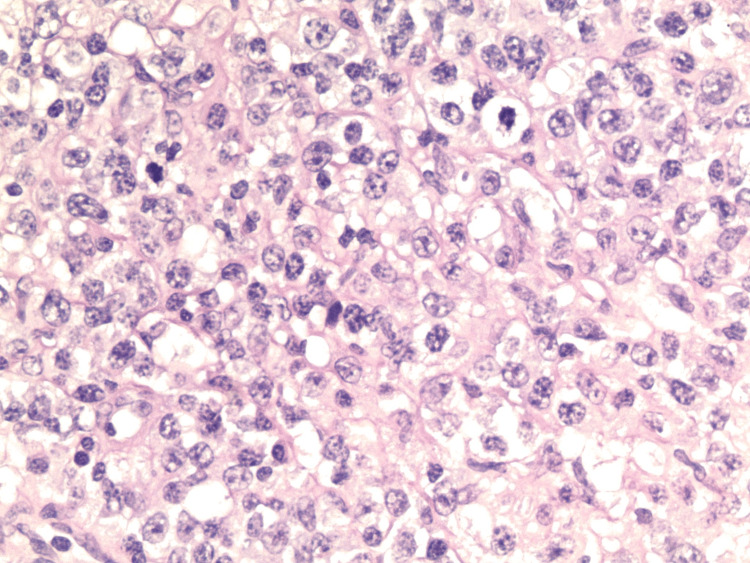
Histopathology of the omental lymph node with H&E stain demonstrating diffuse infiltration of small to medium-sized lymphocytes, plasmoid lymphocytes, and numerous atypical plasma cells, which is consistent with B-cell lymphoma with marked plasmacytic differentiation (400x magnification).

Histopathology of the omental lymph nodes was significant for B-cell lymphoma with marked plasmacytic differentiation (Figures 6, 7). Immunohistochemical stain was positive for CD45, PAX5, CD79a, CD20, BCL6, BCL2, CD138, MUM1, kappa-ISH, and negative for cyclin D1, CD3, CD10, BCL6, and lambda-ISH. The proliferative index measured by Ki-67 was approximately 90%. MYC stain was positive in 10%-20% of the cells. The molecular study was negative for the MYD88 mutation.

After a couple of days of improvement, she once again required increased pressor support and had worsening lactic acidosis. A definitive diagnosis of type B lactic acidosis with underlying B-cell lymphoma associated with systemic lupus erythematosus (SLE) was made, substantiated by the refractory lactic acidosis despite adequate sepsis control and supportive measures. The underlying type B lymphoma with worsened lymphadenopathy, a high proliferative index, and positive MYC staining indicated a high tumor burden and rapid growth, which also supported the diagnosis. Chemotherapy was deferred given the acuity of the condition and the overall poor prognosis. After a discussion with the family, care was transitioned to comfort measures, and the patient passed away shortly after.

## Discussion

The pathophysiology of type B lactic acidosis is commonly described as the Warburg phenomenon. Warburg, in 1923, demonstrated that tumor cells generate excessive lactate via aerobic glycolysis in fully oxygenated environments as opposed to type A lactic acidosis, which occurs in tissue hypoperfusion and hypoxia. This increase in lactate plays a crucial role in all the major steps of carcinogenesis [3]. Aberrant changes in genes like Myc, Ras, P53, and Akt were demonstrated in malignant neoplastic cells, which led to an increase in glycolytic enzymes and glucose transporters, thereby increasing lactate production for the survival of cancer cells [4-6]. Other reasons for lactic acidosis caused by malignancy include cancer tissue hypoxia and reduced hepatic clearance secondary to liver metastasis. Our patient had an aggressive tumor with a high proliferative index, which would lead to high aerobic glycolytic activity. In addition, abnormal liver function along with coagulopathy would have decreased its clearance.

The prognosis of lactic acidosis is governed by the cause, the rate of correction, and the levels of lactate. Mortality has a linear relationship to the duration of lactic acidosis as well as its levels. Type B lactic acidosis was found to be associated with a poor prognosis irrespective of aggressive management, with a reported mortality rate of >90% [7,8].

The first step is identifying the cause. There are various checklists available to determine the cause and tailor the treatment [9]. Initial treatment involves supporting circulation and ventilation with intravenous fluids, blood product transfusion to ensure stable hemoglobin, and maintenance of SpO2 >92%, following which the underlying cause can be corrected. This includes the treatment of sepsis or other causes of hypoperfusion and the removal of the offending agents. When severe and persistent, correction of acidemia with intravenous sodium bicarbonate infusion as well as hemodialysis is encouraged. Continuous renal replacement therapy is preferred over intermittent hemodialysis as it allows for continuous, slow removal of lactate and coadministration of bicarbonate, which decreases the risk of volume overload. Our patient was treated appropriately with these supportive measures, and though there was suspicion of underlying cancer causing lactic acidosis, more readily correctable causes were treated first as per the algorithm. Cytoreductive chemotherapy is the treatment modality for lactic acidosis caused by underlying malignancy, as it is caused by the high tumor burden [8]. Even with cytoreduction, remission is reported only in 10% of patients, and the prognosis remains poor [10].

An approximately three-fold increase in the probability of hematological malignancy was observed in SLE, among which B-cell lymphoma was observed to be the most common. Factors attributing to an increased risk of malignancy include exposure to immunosuppressive drugs, chronic immune dysregulation, SLE-associated autoantibodies, shared genetic susceptibility, and environmental factors [11].

## Conclusions

Type B lactic acidosis caused by underlying malignancy is an oncological emergency. After initial supportive measures, treatment of the underlying cause with modalities including cytoreduction chemotherapy should be initiated promptly when possible. With our case report, we would like to emphasize the need to look for rare and unusual causes when lactic acidosis is refractory to all supportive management to ensure minimal delay in treatment of the underlying cause. This requires an in-depth review of medical history, a thorough physical examination, and medication history.
